# Editorial

**Published:** 1840-04-18

**Authors:** 


### THE MEDICAL EXAMINER.

PHILADELPHIA, APRIL 18, 1840.

### MALINGERING.

In a recent number of the Boston Medical and Surgical Journal, is an interesting letter from Dr. Harlan, containing some particulars of what was supposed to be a case of malingering. The extreme cruelty of an unfounded accusation of the kind, is such, that too much care cannot be exercised to guard against an erroneous opinion in a matter which involves the character of the unfortunate class of individuals who are compelled to resort to public charities.

In military surgery malingering is common, for the purpose of obtaining a discharge, or of avoiding a conscription. There is in these cases something positive to avoid, of a painful nature, and the malingerer is, therefore, strongly tempted to feign disease. But the negative advantages of public charities are so miserable, that very few are willing to feign a disease for the purpose of procuring a miserable pittance or a wretched shelter. With a few rare exceptions, the only malingering that we have seen in an almost constant official connection with medical charities of some twelve or thirteen years duration, is the exaggeration of actual ailments, when the subjects of them are pressed by poverty, or bowed down by mental despondency. This, however, is no more than is met with every day in private practice. The very same disorders which are borne patiently, or are scarcely felt by one individual, are regarded as intolerable grievances by another person when placed under circumstances which produce a different impression upon his mind. This is, to say the most of it, a harmless exaggeration, and one that must be tolerated by true humanity.

But we regret that we have known instances of the contrary kind. We have seen patients labouring under severe, sometimes fatal chronic diseases, regarded as impostors, and we are aware that the same cruel ignorance is sometimes displayed by other practitioners than the learned physician at Paris who could not detect a case of paralysis. The chronic disorders which are thus mistaken or overlooked, are those which, in their nature, are the most obscure, as cancerous affections of the internal viscera, paralysis, diseases of the kidneys, and phthisis. These are readily mistaken, and the physician is sometimes overly suspicious at the expense of a poor wretch who is doomed to a fatal, though insidious disease.

"I attended, this morning, an interesting consultation at the hospital La Charité, in the Salle St. Joseph, under the direction of M. Cruvelhier. The case was that of an English woman, who, with her husband, had for some years resided in Paris, and chiefly, under the plea of some disease or other, at the expense of an English benevolent society. She finally, some months since, came under the care of Dr. Macgloughlin, one of the visiting physicians of the Society ; the patient, at this time, displaying most of the symptoms of paraplegia of the inferior, and hemiplegia of the superior portions of the body. From the previous history or the case, and certain irregularities in the diagnostics, Dr. M. considered the patient as an impostor, assuming disease with the view of imposing on the charity of her compatriots. The benevolent society, in consequence of this report of its official agent, suppressed all further contributions. In this stage of the proceeding, M. Cruvelhier was requested to take charge of the patient. He not only entirely disagreed in opinion with Dr. M., but gave the husband of the patient a written testimony that the case was really one of total paralysis of the lower portions of the body, and of the right arm and right side of the face. With this high authority on his side, the husband, (Mr. Harding) appealed to the public, and Dr. M. was posted as a cruel ignoramus, who had voluntarily robbed a poor decrepid female of the last means of support. The doctor, in self-defence, commenced legal proceedings against the calumniator, and gained his suit—his opponent being fined and imprisoned. M. Cruvelhier now took the patient to his ward in the hospital, and called, in consultation, all the medical faculty of the institution, who, one and all, on minute examination, entertained the same opinion as M. Cruvelhier, that the patient really laboured under a general and incurable paralysis.

These opinions being made public by the numerous students attending the practice of the ward, occasioned considerable excitement and discussion among the profession—when, finally to settle the matter, the medical faculty of the hospital offered to Dr. M. a public examination of the patient, before his medical friends, the students and profession in general.

Accordingly, this morning, at 9 o'clock, the appointed hour, the ward of M. Cruvelhier was crowded to overflowing. M. Velpeau, making his appearance last, found it almost impossibe to force his way into the circle which was attempted to be formed around the patient's bed, which had been removed to the middle of the room. M. Cruvelhier, observing his efforts, interrupted the discussion by crying out, 'Voici Mons. Velpeau.' Mr. V. immediately replied, 'I fear that I shall be Mons. *Sans*peau before I attain the circle.' The French, by the way, are great punsters.

The discussion was opened by Mons. Cruvelhier, who gave a lucid and candid history of the whole case, and repeated his opinion, in the most decided manner, of the truth of the diagnosis that he and his confrères had already announced. The patient, whose appearance indicated that the approach of dissolution was not many days distant, was subjected to every kind of examination and experiment, in order to test the validity of her symptoms—Mons. Cruvelhier repeating, continually, 'Show me the power of motion—prove to me the existence of sensibility.' To me it appeared evident, that a partial or slight examination of the patient in the present state of the case, was sufficient to diagnosticate the existence of paralysis, though probably not to the extent affected by the miserable patient—who, no doubt, simulated symptoms even now, in a dying state—the 'malade immaginaire,' the simulator of disease, had become the real victim.

After two hours of torture to the patient, M. Cruvelhier remarked to Dr. M., 'You are obliged to confess that you are unable to detect either the power of motion or the existence of sensibility in the muscles affected, and it now becomes your duty, as a man of honour, a gentleman, and a respectable practitioner, as you are, to confess your error, and give the parties a certificate accordingly.' When Dr. M. declined to do this, hisses proceeded from the medical class. Dr. M. becoming irritated, declared, striking his fist against his hand, 'I'll challenge the whole of you,' and the class immediately separated amidst loud peals of laughter.

The most convincing proof of the existence of paraplegia, consisted in the introduction of the catheter, the bladder being full; a small quantity of urine began to flow, but immediately ceased—and was only voided by pressure on the abdomen. Dr. M. doubts the fairness of the manner in which this experiment was made."

